# MicroRNA Profile Alterations in Parathyroid Carcinoma: Latest Updates and Perspectives

**DOI:** 10.3390/cancers14040876

**Published:** 2022-02-10

**Authors:** Marta Wielogórska, Beata Podgórska, Magdalena Niemira, Małgorzata Szelachowska, Adam Krętowski, Katarzyna Siewko

**Affiliations:** 1 Department of Endocrinology, Diabetology and Internal Medicine, Medical University of Bialystok, 15-276 Bialystok, Poland; bbpodgorska@gmail.com (B.P.); mszelachowska@poczta.onet.pl (M.S.); adamkretowski@wp.pl (A.K.); katarzynasiewko@o2.pl (K.S.); 2 Clinical Research Centre, Medical University of Bialystok, 15-276 Bialystok, Poland; magdalena.niemira@umb.edu.pl

**Keywords:** parathyroid carcinoma, miRNAs, biomarkers, miRNA expression regulation

## Abstract

**Simple Summary:**

Despite the considerable development of diagnostic tools, distinguishing between benign and malignant parathyroid tumors poses a significant diagnostic challenge. Epigenetic regulations, including noncoding microRNAs (miRNAs), have recently emerged as a new and promising source of biomarkers. MiRNAs are post-transcriptional regulators of gene expression. These tissue-specific molecules are known to be deregulated between cancer and normal cells. This review delineates changes in miRNA expression in parathyroid carcinoma (PC), advancing our understanding of PC tumorigenesis and emphasizing, at the same time, that miRNAs can be further exploited for diagnostic and therapeutic purposes.

**Abstract:**

Parathyroid tumors are a genetically heterogenous group with a significant variability in clinical features. Due to a lack of specific signs and symptoms and uncertain histopathological criteria, parathyroid carcinomas (PCs) are challenging to diagnose, both before and after surgery. There is a great interest in searching for accurate molecular biomarkers for early detection, disease monitoring, and clinical management. Due to improvements in molecular pathology, the latest studies have reported that PC tumorigenesis is strongly linked to the epigenetic regulation of gene expression. MicroRNA (miRNA) profiling may serve as a helpful adjunct in distinguishing parathyroid adenoma (PAd) from PC and provide further insight into regulatory pathways involved in PTH release and parathyroid tumorigenesis. So far, only a few studies have attempted to show the miRNA signature for PC, and very few overlaps could be found between these relatively similar studies. A global miRNA downregulation was detected in PC compared with normal glands among differentially expressed miRNAs. This review summarizes changes in miRNA expression in PC and discusses the future research directions in this area.

## 1. Introduction

Parathyroid glands are endocrine organs that regulate calcium homeostasis by releasing parathormone (PTH) [1]. They sense the extracellular calcium concentrations through a G-protein-coupled calcium-sensing receptor (CASR) expressed on parathyroid cell membranes [2,3]. The inositol-triphosphate (IP3) intracellular pathway suppresses PTH release and synthesis in response to CASR activation with an increased extracellular calcium concentration [1,4]. The excessive secretion of PTH is a common disorder known as hyperparathyroidism. 

Primary hyperparathyroidism (PHPT) is the third most frequently reported endocrine disorder, following diabetes and thyroid disorders, with a prevalence of 0.1–0.4% in the general population and 2–4% in post-menopausal women [5,6,7]. The prevalence of PHPT is estimated to have escalated in recent years, probably due to the increase in serum calcium and PTH measurement [8]. PHPT is caused by parathyroid adenoma (PAd) (80–85%), parathyroid gland hyperplasia (PHyp) (10–15%) and parathyroid carcinoma (PC), which occurs in about 1–5% of cases of PHPT [8,9]. Parathyroid tumors were considered largely monotypic entities arising from the clonal expansion of a single transformed progenitor [10]. The latest studies provide information on how highly heterogenous they are [8,11]. 

Several publications describe atypical PAds as another subset of parathyroid proliferative disorders occurring in 1% of PHPT cases. Atypical adenomas present aggressive features that are either clinically or histologically more serious than those of typical adenomas. However, they do not meet the requirements for PCs according to the histopathological criteria defined by the World Health Organization (WHO) [1,12].

PC is a rare endocrine malignancy accounting for 0.005% of all cancers. However, the first nationwide study of PC in Asia demonstrates an increase in the incidence of PC, similar to trends in Western countries [8,11]. Patients with PC usually present a long clinical history complicated by tumor recurrences and progressive end organ damage [13,14,15]. Clinical symptoms such as renal failure, bone disease, cardiac arrhythmia, or neurocognitive dysfunction are mainly related to excess PTH secretion and intractable hypercalcemia [12,16,17,18,19]. The prognosis in PC is poor to moderate, and most patients with PC require multiple surgeries [14,15]. At 5-year follow-up, the survival rate is 76 to 85% in Western populations [20,21], whereas the 5- and 10-year survival rates estimated in 2021 by Kong in the Korean population were 86.5% and 72.9%, respectively [8]. Despite the development of diagnostic tools, there are observed cases of patients with the presumed benign disease diagnosed with PC after developing local recurrences. In the study by Wang et al., misdiagnosis occurred in >80% of the patients with PC before the operation [15]. The same observation was presented by Kong et al. in their study, in which they emphasized the value of preoperative suspicion of the disease [8]. 

In this regard, the search for accurate biomarkers for early detection, disease monitoring, and risk assessment is a priority for a correct diagnosis. It remains one of the clinical challenges in precisely distinguishing PC and PAd.

## 2. MicroRNAs as a Potential Source of Biomarkers in Parathyroid Tumors

MicroRNAs (miRNA, miR) are short, 17-nucleotide- to 25-nucleotide-long, non-coding RNAs [22,23,24]. They are regulators of gene expression at the post-transcriptional level [25,26]. A single miRNA may regulate the expression of more than a hundred different messenger RNA (mRNAs) targets, and each mRNA can be targeted by several hundred different miRNAs [27,28,29]. MiRNAs negatively regulate gene expression by base-pairing to partially complementary sites on mRNAs, usually in the 3′ untranslated region (UTR) [30,31]. 

MiRNAs can be detected in various body fluids, such as plasma, serum, amniotic fluid, bronchial lavage, breast milk, cerebrospinal fluid, peritoneal fluid, pleural fluid, saliva, seminal fluid, tears, and urine. Circulating miRNAs are remarkably stable in the blood and seem more practical in diagnostic use than tissue miRNA expression. They can serve as an informative biomarker for detecting various cancers and other diseases [21,23,32,33]. Circulating miRNAs comprise free and vesicular miRNAs [34]. Currently, circulating miRNA measurement has not yet been widely used in clinical practice [35]. However, more and more potential biomarkers to establish diagnostic and prognostic information have been provided in recent years. 

Titov et al. created a diagnostic panel to enable the identification of parathyroid and thyroid tumors comprising the analysis of the expression of selected miRNAs (miR-146b, miR-221, miR-375), mRNA expression (*HMGA2, GCM2*), the V600E mutation in the *BRAF* gene and mitochondrial-to-nuclear DNA ratio. The expression level of the *GCM2* gene (glial cells missing homolog 2, a zinc finger transcription factor) was used as a marker for the detection of parathyroid cells. However, PCs were not analyzed in their study [36].

## 3. Genetic Background of Parathyroid Tumors

Most cases of hyperparathyroidism are sporadic, but there are also some familial syndromes representing approximately 5–10% of parathyroid tumors in PHPT: multiple endocrine neoplasia type 1 (MEN 1; Online Mendelian Inheritance in Man, OMIM #131100) and 2A (MEN 2A; OMIM #171400), familial isolated hyperparathyroidism (OMIM #145000), hyperparathyroidism–jaw tumor syndrome (HPT-JS; OMIM #145001), and familial hypocalciuric hypercalcemia (HHC1; OMIM #145980), (HHC2; OMIM #145981), (HHC3; OMIM #600740) [37]. PHyp might be associated with inactivating mutations of the oncosuppressor *CDKN1B*, coding for the cyclin-dependent kinase two inhibitors (p27Kip1) protein involved in the occurrence of cell cycle control, and might be a part of multiple endocrine neoplasia type 4 (MEN4; OMIM #610755) [38]. The loss of tumor suppressor genes rather than the gain of oncogenes is mainly related to parathyroid tumorigenesis. It is noteworthy that genes involved in developing familial parathyroid tumors are also associated with parathyroid sporadic tumors [39,40]. 

There is a lack of histopathological features that may help reliably distinguish sporadic and hereditary parathyroid tumors [41]. In their study in 2018, Hwang et al. identified miR-199-5p as differentially expressed miRNA between sporadic and hereditary parathyroid tumors, with 67% sensitivity and 100% specificity for distinguishing these two types of tumors. MiR-199b-5p was significantly downregulated and negatively associated with PTH levels in sporadic tumors, whereas the hereditary tumors were connected with its upregulation [42].

PCs might be caused by anomalies in genes, such as cell division cycle 73 (*CDC73), MEN-1*, retinoblastoma 1 (*RB*), tumor protein p53 (*TP53*), cyclin D1 (*CCND1*), the enhancer of zeste 2 polycomb repressive complex 2 subunits (*EZH2*), adenomatous polyposis coli (*APC*), glycogen synthase kinase 3 beta (*GSK3B*), and prune homolog 2 (*PRUNE2*) [17].

*CDC73* maps to 1q31.2 and is considered one of the key genes in the pathogenesis of PC. Its mutation occurs in most patients with HPT–JT syndrome and 50–70% of patients with sporadic PCs [12,16,43,44,45]. Up to 15% of PC patients can be diagnosed with HPT–JT syndrome [13]. 

Parafibromin is a 531 amino acid protein encoded by the *CDC73* oncosuppressor gene. The interaction between parafibromin and β-catenin regulates various cellular processes involved in tumorigenesis [13,46,47,48,49,50]. The absence of parafibromin staining might be an adjunct immunohistochemical marker in the clinical routine for distinguishing benign from malignant parathyroid tumors, predicting the clinical outcome and mortality rate even better than *CDC73* mutation [51,52,53]. 

## 4. MicroRNA Profile Alterations in Parathyroid Carcinomas

Normal cellular homeostasis is maintained by a series of control mechanisms, including the precise control of miRNA levels. MiRNAs can modulate hormone synthesis and secretion by CASR expression, and the initiation and progression of endocrine tumorigenesis. Genome-wide profiling revealed that miRNA profile alterations are linked to tumor type, tumor grade, and clinical outcomes [30,54]. 

### 4.1. Differentially Expressed Tissue-Related microRNAs in Parathyroid Carcinoma

For the first time, Corbetta et al. suggested the existence of an altered miRNA expression pattern in PCs. In summary, they profiled 362 miRNAs through a real-time qPCR in four PC and two normal parathyroid glands. All PC samples were sequenced to indicate CDC73/HRPT2 mutations. Fourteen significantly downregulated miRNAs and three significantly upregulated miRNAs were identified in the PCs. Four differentially expressed miRNAs (miR-139-3p, miR-296-5p, miR-222, and miR-503) were the most markedly deregulated between PC and the normal parathyroid gland. Malignant parathyroid tissue overexpressed miR-222 and miR-503 when compared with normal glands. MiR-296 and miR-139 were underexpressed in malignant tissue [55]. 

The identified miRNA signature was a subject of further analysis in a subset of sporadic PAds. Only miR-296, miR-222 and miR-503 differed significantly between PC and PAd. MiR-222 and miR-503 were overexpressed in PCs concerning Pads, and miR-296 was downregulated in PCs. There were no significant differences in the levels of miR-139 between PCs and PAds [55]. The three most different miRNAs between PC and PAd (miR-222, miR-503 and miR-296) were used to obtain a computed miRNA score for the diagnosis of PC [55]. Unfortunately, its efficacy in distinguishing PC from PAd in further studies was not satisfying, probably due to a limited number of samples and the genetic heterogeneity of PCs and PAds [12]. 

**Figure 1:**
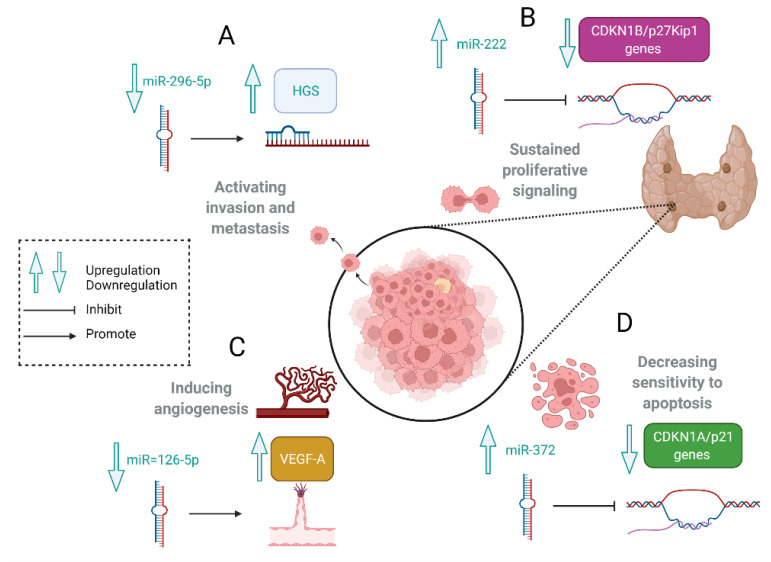
The mechanisms of roles of selected miRNAs involved in parathyroid tumorigenesis. (**A**). MiR-296 negatively correlates with HGS mRNA expression levels. HGS overexpression leads to tumor cell invasiveness and metastasis according to the downregulation of E-cadherin. (**B**). MiR-222 inhibits *CDKN1B/p27Kip1*  involved in cell cycle regulation. (**C**). The downregulation of miR-126-5p induces angiogenesis via VEGF. (**D**). MiR-372 inhibits *CDKN1A/p21* genes and decreases parathyroid cells’ sensitivity to apoptosis. Created with BioRender.com (accessed on 1 February 2022).

The hepatocyte growth factor receptor-regulated tyrosine kinase substrate (HGS) mRNA is a direct target of miR-296. Its overexpression leads to crucial cancer-promoting processes such as tumor cell invasiveness and metastasis according to the downregulation of E-cadherin responsible for maintaining epithelial integrity. The perturbation of cell polarity and integrity in epithelial cells is considered one of the earliest phenotypical changes in cellular transformation, heralding invasive cancer. Much higher levels of HGS mRNA were observed in PCs than those recorded in PAds and parathyroid normal glands. It might indirectly suggest that miR-296 is involved in parathyroid cell carcinogenesis. MiR-296-5p is progressively downregulated during tumor progression. This process is considered to be correlated with metastatic disease in not only PCs but also colorectal, breast, liver, bile ducts, gastric, and lung carcinomas [1,55,56,57]. The mechanisms of roles of selected miRNAs involved in parathyroid tumorigenesis are shown in Figure 1.

Overexpression of miR-222 is reported in malignant parathyroid tumors. P27 is a cyclin-dependent kinase inhibitor (CKI) targeted by this molecule. P27 inhibits the activity of cyclin/cdk complexes during G0 and G1 and acts as a negative regulator of the cell cycle. The downregulation of p27 is a critical event for the G1/S transition and contributes to parathyroid tumorigenesis (Figure 1) [55,58]. 

Rahbari in another study compared PC with Pad, primary PHyp, and normal parathyroid glands. Authors identified 167 miRNAs significantly dysregulated in PCs compared with normal tissue [16]. A total of 91 miRNAs were differentially expressed between PAds and PCs. The miRNA profiles detected in PHyp showed the upregulation of many miRNAs, whereas, in PCs, most of the miRNAs were downregulated. The expression levels of miR-126-5p, miR-30b, and miR-26b were reported significantly differentially in PCs compared with PAds [12,16,54,55]. MiR-126-5p was the best discriminator of PC from Pad, according to a receiver-operating characteristic curve (ROC) analysis with an area under the curve (AUC) of 0.776 [16]. The presented subset of candidate miRNA markers in PC was inconsistent with Corbetta’s study [55].

MiR-30b and miR-26b are involved in cell mortality processes, and miR-126-5p is considered as an endothelial-specific miRNA that acts as a tumor suppressor in various types of human cancer. MiR-126-5p targets genes involved in oncogenesis such as *PI3K*, *KRAS*, *EGFL7*, *CRK*, *ADAM9*, *HOXA9*, *IRS1*, *SOX2*, *SLC7A5*, and *VEGF*, and its low expression induces cancer cell proliferation, migration, and invasion (Figure 1) [59,60,61].

Vaira et al. checked the expression profile of a subset of miRNAs belonging to C19MC and the miR-371–373 clusters in a series of normal parathyroid glands, adenomas, carcinomas, and distant metastatic lesions. The amplification of C19MC with miR-517c upregulation was observed in most PCs. Its expression was positively correlated with serum calcium, PTH and tumor weight. The median expression levels of miR-519d, miR-518e, miR-517c, and miR-371 in PCs were comparable with those in human placentas. The most significant difference between PC and Pad was marked in miR-517c expression. Additionally, the aberrant expression of miR-519d, miR-520g, miR-518e, and miR-372 positively correlated with serum PTH levels, whereas miR-520d, miR-520g, and miR-372 positively correlated with serum calcium levels [62].

All presented data suggest an oncogenic role of the mentioned clusters in parathyroid tumorigenesis [62]. The miRNA cluster C19MC maps on chromosome 19q13.4 and consists of 46 genes encoding 56 mature miRNAs. It is considered to be the largest human-specific miRNA gene cluster that is silenced in most adult normal cells by hypermethylation. Typically, its embryonic expression pattern occurs in embryonic undifferentiated cells, and their differentiation leads to their rapid downregulation. Its expression increases significantly in trophoblasts from the first to the third trimester of gestation, and the human placenta is the only human adult tissue that physiologically expresses C19MC miRNAs. The C19MC miRNA cluster is involved in tumor invasion and metastasis, and its re-expression was noted in several human tumors. It may provide a new explanation of parathyroid tumorigenesis with activated embryonic transcription factors and signaling pathways involved in embryogenesis. In particular, the loss of promoter methylation at the C19MC cluster, present in half of the parathyroid tumors, correlates with carcinomas and metastatic disease [1,54]. 

In 2018, Verdelli presented a study in which the results revealed an aberrant expression of miR-372 in half of the PAds and most atypical adenomas and PCs. In the authors’ opinion, this molecule is used by parathyroid tumor cells to increase PTH synthesis, deregulate the Wnt/β-catenin pathway via the upregulation of the Wnt-inhibitor, Dickkopf 1(DKK1), and partially decrease sensitivity to apoptosis due to p21 repression (Figure 1) [63]. 

The most common sites for PC metastases are the lungs, liver, and bones. In their epidemiological study, Sandelin et al. reported distant metastases in 25% of patients with PC [46,64]. The increased expression of C19MC miRNAs and miR-372 was documented in five PC metastases. It indicates that alterations in the miRNA profile observed in PCs could be identified with a similar pattern of expression in their distant metastasis [13,62]. 

With the growing need to evaluate all putative prognostic markers, Hu et al. constructed a study using nine candidate miRNA markers identified by prior studies. All of them were validated by qRT-PCR and assessed in a Chinese study group with PCs. Interestingly, PCs account for 5–7% of PHPT cases in China, this proportion is much higher than in Western populations. MiRNA correlations with clinical and pathologic features were also considered. Taken together, the expression profile of miR-139, miR-222, miR-30b, miR-517c and miR-126 differed considerably between PCs and PAds. Similar observations regarding miR-139 and miR-122 have been initially demonstrated in Corbetta’s study, but Rahbari did not confirm this data [12]. In contrast to Vaira’s study, the expression level of miR-517c was lower in PCs than in PAds in a study of the Chinese population. MiR-30b was negatively correlated with PTH and serum calcium levels in PCs. Additionally, to provide the best chance of detecting PCs, the logistic regression analysis of data provided by Rahbari was used. This method presented a combination of miR-139 and miR-30b, with a calculated AUC of 0.888, as the most promising marker for diagnosing PC. Unfortunately, differentiating malignant and benign lesions was not possible using Corbetta’s miRscore in this cohort. As with the majority of studies, the design of this study is subject to limitations. The gene sequencing of *CDC73* in a fraction of a study cohort and the lack of long-term follow-up are the main limitations in Hu et al.’s study. A sample size that is too small reduces the power of the study, and the authors have also considered it to be a limitation in their study. However, they have presented the highest number of PC cases compared with the rest of the proposed studies so far [12]. In Table 1, we have summarized studies concerning the expression of miRNA in PC.

### 4.2. Differentially Expressed Serum-Related microRNAs in Parathyroid Carcinoma

Free and vesicular miRNAs are important components of liquid biopsy. One of the latest studies published in 2021 presented the results of comparing the miRNA expression profile in serum exosomes in PCs and PAds. The study group comprised four patients with PC and four patients with PAd. As in the studies presented previously, the study’s main limitation was a small sample size of PC. MiR-27a-5p was upregulated in PC serum and indicated by authors as a putative tumor marker for the preoperative identification of patients with PC (Table 1) [34]. MiR-27a-5p activates the Wnt/β-catenin signaling pathway and plays a vital role in the epithelial-mesenchymal transition (EMT) [65]. Wnt signalling regulates the expression of cyclin D1 involved in parathyroid neoplasia. The recent studies provide new information indicating the association of dysregulated WNT/β-catenin signalling and the accumulation of active non-phosphorylated β-catenin with the pathogenesis and progression of PC [66,67]. In the largest genomic sequencing study of PC, Pandya et al. identified the first sporadic PC with somatic mutations in the Wnt canonical pathway [43].

In another study, Krupinova examined serum samples of 13 patients with PC and 11 patients with PAd. A total of 17 miRNAs were differentially expressed between PC and PAd. Among them, miR-342-3p, downregulated in PC, was the most promising biomarker in distinguishing patients with PC and Pad, according to a ROC analysis with an AUC of 0.888 (Table 1). None of the studies mentioned earlier found a difference in miR-342-3p expression. According to the latest studies, miR-342-3p plays a role in the development of various tumors and negatively regulates cell viability. Moreover, its potential as a therapeutic target in non-small cell lung neoplasms is considered [47,68].

### 4.3. Identification of miRNA Alterations in PC-Studies Limitations

Although the role of miRNAs has been studied in different tumor types, only a few published original studies have so far attempted to compare miRNA expression signatures in tissues and serum between malignant PC and benign PAd. According to presented studies, the expression of a series of miRNAs, including miR-296, miR-222, miR-503, miR-126, miR-26b, miR-517c, miR-30b, miR-139, miR27, and miR-342, was documented to be different between PC and PAd. Table 2 summarizes differentially expressed miRNAs in PCs. Nevertheless, the results were inconsistent across studies. The profile of miRNAs was usually assessed by quantitative polymerase chain reaction (qPCR)-based arrays comparing miRNA expression profiles in cancer tissues and serum with those observed in benign adenomas to find differentially expressed transcripts [54]. 

The principal limitations of the studies were their restricted sample sizes due to the rarity of PC. Other limitations were the different types of normal controls included in the analyses and the genetic heterogenesis of parathyroid tumors that might not have always been taken into account. Furthermore, the different platforms used (qPCR and array-based technique) by other researchers might have provided miRNA expression data that were difficult to compare and generated controversial results [69]. Several studies showed a lack of concordance between different methods when using the same sample source. Therefore, precautions must be taken in the initial study design phase during planning the cohort composition since correlations between miRNA levels and age, sex, and ethnicity were demonstrated [35]. 

### 4.4. Familial Syndromes and Parathyroid Tumors

MEN-1, also known as Wermer syndrome, is a rare hereditary tumor syndrome (prevalence 1:30,000) inherited in an autosomal dominant pattern. It is associated with not only multiple endocrine neoplasms primarily of the parathyroid, gastroenteropancreatic tract, neuroendocrine cells, and the anterior pituitary, but also nonendocrine manifestations including meningiomas, ependymomas, lipomas, angiofibromas, collagenomas, and leiomyomas [70]. Although MEN1-related neuroendocrine tumors can be malignant, parathyroid lesions are usually benign hyperplasia, and PCs have occasionally been reported [13]. MEN-1 is associated with loss-of-function mutations of the tumor suppressor gene *MEN1,* encoding menin. Due to the decreased life expectancy in patients with MEN-1, early genetic and clinical diagnosis is crucial [13,70,71,72]. 

The role of epigenetic factors, including miRNA deregulation in MEN-1 tumorigenesis, was suspected due to an individual clinical phenotype even in patients with the same MEN-1 mutation. MiR-24-1-5p, belonging to the cluster miR-23b, targets an essential protein for osteoblast maturation, called Smad5, and the *MEN1* gene [1,13,73,74]. The analysis of miR-24 expression revealed that its presence is observed only in MEN-1-related PAd that conserved the wild-type MEN-1 allele, and there is no expression of miR-24 in patients that lost both *MEN1* alleles [73]. Studies evaluating the role of miR-24 in MEN1-related parathyroid neoplasia revealed the presence of an autoregulatory network between miR-24 and the menin protein, based on an increasing miR-24 level in response to the overexpression of menin, which in sequence suppresses *MEN1* gene expression [1,54,73,75,76]. In 2016, Luzi et al. presented a novel mechanism revealing that menin binds specifically to the primary RNA sequence pri-miR-24-1 and facilitates the processing of its specific miRNA at the level of pri-miRNA processing. The study was performed in the BON1 cell line derived from the lymph node metastasis of a pancreatic neuroendocrine tumor [77]. According to this information, miR-24-1 may represent a promising target to develop a therapeutic silencing antagomir restoring the correct expression of menin to control MEN1 tumorigenesis progression and prevent the development of cancers in the target tissues [75]. The rarity of PC in MEN-1 syndrome might be connected with the miR-664-targeting *CDC73* gene and its downregulation in PAds with the biallelic loss of the wild-type *MEN*1 gene [78].

## 5. Latest Updates and Future Perspectives

MiRNAs have been described in a large number of studies to have an important role in a vast range of biological processes such as proliferation, differentiation, and apoptosis. Their expression is specific to tissue type. We reviewed data about a series of candidate miRNA markers that might be used to increase the accuracy of PC diagnosis, help identify patients for closer follow-up or immediate reoperation with more radical resection, and help reveal new therapeutic approaches by developing novel molecular mechanisms and miRNA-targeted therapies for the cure of parathyroid tumors. A subset of specific miRNAs that discriminate PC from PAd is more clinically relevant than differentiating PC from normal parathyroid glands. The latest studies have better defined the genomic landscape of PCs and revealed significant progress toward a complete molecular characterization of this neoplasia.

Most of the deregulated miRNAs in PC are repressed, whereas most miRNAs are upregulated in PHyp. MiRNA profiling may serve as a helpful adjunct to distinguish malignant from benign lesions and elucidate regulatory pathways involved in PTH release and parathyroid tumorigenesis. Unfortunately, there are some limitations to discovering genes regulated by selected miRNAs according to the lack of suitable parathyroid cell lines. In vitro parathyroid cell models are almost not useful for transfection studies. However, numerous identified miRNAs have gene targets validated in other cell systems [1,16,54,79].

In a study by Hu, miR-30b was negatively correlated with PTH and serum calcium levels in PCs. Therefore, this molecule should be further analyzed for a novel therapeutic target for PC or severe hypercalcemia [12]. MiR-296-5p, miR-222 and miR-126 are also differentially expressed miRNAs in PCs. They are identified as the principal regulator of cancer stem cells (CSC) occurring in many neoplasms. CSCs have the ability of self-renewal, and can be involved in tumorigenesis and tumor recurrence. Because regeneration and recurrence significantly hamper the efficacy of conventional cancer treatment, interactions between miRNA regulation and CSCs might be a clue to new targeted therapies [80]. Moreover, CDC73, one of the critical genes involved in the pathogenesis of PC, has been reported to be downregulated by oncogenic miR-155. In 2013, Rather suggested that restoring CDC73 levels by using a miR-155 inhibitor may lead to decreased cell proliferation and enhanced apoptosis, and may have an important role in the therapeutic intervention of cancers [45].

Liquid biopsy has been gaining ground as a promising diagnostic tool. This non-invasive method helps to reduce discomfort and risk to patients. MiR-27a-5p is upregulated in PC serum exosomes and might be a putative tumor marker for the preoperative identification of patients with PC [34]. 

MiR-7 has attracted the attention of endocrinologists with its involvement in the initiation and progression of various endocrine-related neoplasms. It has been considered a tumor-suppressive role and reduces the growth of adrenocortical carcinomas [81]. Unfortunately, studies that have been published so far have not confirmed the differential expression of miR-7 between PC and normal parathyroid glands [82].

Further studies and enormous research efforts are still necessary to better understand the role of miRNAs in PCs. All presented studies have aroused huge interest, and significant advancements are expected in the following years. It is hoped that high-throughput molecular methods will benefit PC diagnostics and treatment.

## 6. Conclusions

MiRNA regulation in parathyroid tumors remains poorly recognized. There is still a long way to go before a real clinical application. Only a few studies have attempted to show the miRNA signature for PC, and very few overlaps could be observed across relatively similar studies. Taken together, a global downregulation of miRNAs was documented in PC compared with normal glands among differentially expressed miRNAs. Among the downregulated miRNAs are miR-126, miR-26b, miR-30b, miR-296, and miR-139. Upregulated miRNAs comprise miR-503, miR-222, and miR-27a. The same limitations, such as the scarcity of PC patients with a high risk of underestimating potentially useful markers, have appeared in subsequently analyzed studies. Furthermore, the series of miRNA candidate markers should be examined in parathyroid neoplasia characterized for the genetic status of *MEN1* and *HRPT2/CDC73* because miRNA might interact differently according to the genetic background. When researchers choose the technique used in their study, they should be aware of their limitations, and the same method should always be used when the results are supposed to be compared. Emerging evidence has shown that even the partial inactivation of tumor suppressors can significantly contribute to tumorigenesis. In conclusion, all of the presented data are promising, but all of them require validation in an independent and larger cohort to exclude discordant results. Interestingly, studies checking associations between miRNAs and aggressive clinical features in PCs have not been performed yet. The field of research for reliable prognostic markers in PCs remains open. The potential use of circulating miRNAs and tissue as diagnostic biomarkers and therapeutic targets requires further investigation.

## Figures and Tables

**Table 1:** Summary of studies looking at miRNA expression in PC.

Aim of the Study	Sample Type	Sample Size	Method	miRNA Expression	Main Findings	Limitations	Others	References
The identification of differentially expressed miRNAs in PCs compared with normal tissues	Tissue	4 PCs 26 PAds 3 PNs	Microarray, qPCR	miR-296 ↓ in PCs vs. PN and PAd miR-139 ↓ in PCs vs. PN and PAd miR-503 ↑ in PCs vs. PN and PAd miR-222 ↑ in PCs vs. PN and PAd	Global miRNA profiles correctly sorted PCs from PNs miR-296, miR-139, miR-222, and miR-503 were significantly differentially expressed between PCs and PNs PCs could be discriminated from PAds by a computed score based on the expression levels of miR-296, miR-222, and miR-503 The potential role of miR-296 as an oncosuppressor in PCs	The small sample size analyzed in the study	All PC samples were sequenced to indicate CDC73/ HRPT2 mutations	Cobetta et al., 2010 [55]
Checking if parathyroid neoplasm had a distinct miRNA signature	Tissue	9 PCs 12 PAds 15 PHyps 4 PNs	miRNA arrays, qPCR	miR-126 ↓ in PCs vs. PAd miR-26b ↓ in PCs vs. PAd miR-30b ↓ in PCs vs. PAd	miR-126, miR-26b, and miR-30b were significantly different between PAd and PC miR-126 levels were the most accurate differentiator between PC and PAd (AUC 0.776) Most miRNAs were downregulated in PCs Most miRNAs were upregulated in pHyps	The lack of the genetic characterization of PCs The small sample size analyzed in the study	Primary PHyp was analyzed in the study	Rahbari et al., 2011 [16]
An assessment of the expression of C19MC–MiR371–3 clusters in parathyroid tumors	Tissue	15 PCs+5 matched mts 24 PAds 6 PNs	qPCR	miR517c ↑ in PCs vs. PAds	C19MC cluster aberrations are a characteristic of PCs with respect to PAds miR-517c were the most significantly different in expression between PCs and PAds miR-517c positively correlated with serum calcium, PTH and tumor weight The copy number variations of 19q13.4 loci were associated with miR-517c up-regulation	The small sample size analyzed in the study	The set of miRNAs was chosen according to their genomic location and biological importance PC samples were sequenced to indicate CDC73/ HRPT2 mutations	Vaira et al., 2012 [62]
The verification of a group of miRNA markers in a new series of samples to explore their potential significance in PC diagnosis	Tissue	17 PCs 41 PAds	qPCR	miR-222 ↑ in PCs vs. PAds miR-139 ↓ in PCs vs. PAds miR-126 ↓ in PCs vs. PAds miR-30b ↓ in PCs vs. PAds miR-517c ↓ in PCs vs. PAds	miR-139, miR-222, miR-30b, miR-517c, and miR-126 were differentially expressed between PCs and PAds The combination of miR-139 and miR-30b was the best diagnostic marker between PCs and PAds (AUC 0.888) miR-30b was negatively correlated with serum calcium, PTH and akaline phophatase	*CDC73* gene sequencing was completed in only a fraction of the PCs The diagnosis of PCs was histopathologically established, but local recurrences or metastases were not observed during follow-up	The study design was based on the validation of nine candidate miRNA markers, identified by prior studies in a new set of PC cases from the Chinese population	Hu et al., 2018 [12]
The investigation of the differences in the miRNA expression profile present in serum exosomes by comparing PC and PAd	Serum	4 PCs 4 PAds	NGS, qPCR	miR-27a ↑ in PCs vs. PAds	miR-146b-5p, miR-27a-5p, miR-93-5p, miR-381-3p, and miR-134-5p were differentially expressed in PC patients The expression of exosomal hsa-miR-27a-5p was significantly different between PCs and PAds (AUC 0.8594) and it could be a valuable molecular marker for PC diagnosis	The small sample size analyzed in the study The lack of the genetic characterization of PCs	It is the first study investigating the serum exosomal miRNA in patients with PC	Wang et al., 2021 [34]
Comparing the serum miRNA expression alterations between patients with benign and malignant parathyroid tumors	Serum	13PCs 11PAds	qPCR	miR342-3p ↓ in PCs vs. PAds	miR-342-3p was the most promising biomarker in distinguishing patients with PC and PAd (AUC 0.888)	The found miRNA biomarker is not specific only for PC The correlation between miRNAs, calcium and PTH concentrations in the two examined groups was not excluded	Study evaluating serum miRNA expression profiles	Krupinova et al., 2021 [47]

Parathyroid adenoma (PAd); Parathyroid carcinoma (PC); Parathyroid hyperplasia (PHyp); Normal parathyroid glands (PN); Metastasis (Mts); Next-generation sequencing (NGS); Quantitative polymerase chain reaction (qPCR); Decrease in miRNA expression levels (↓); Increase in miRNA expression levels (↑); Area under the receiver-operating characteristic curve (AUC).

**Table 2:** Differentially expressed miRNAs in PCs.

miRNA	MiRBase	Chromosome	Variation in PCs vs. PAds	Sample Type	References
miR-126	hsa-miR-126-5p	9q34.3	↓	Tissue	Rahbari et al. 2011 [16] Hu et al., 2018 [12]
miR-26b	hsa-miR-26b-5p	2q35	↓	Tissue	Rahbari et al. 2011 [16]
miR-30b	hsa-miR-30b-5p	8q24.22	↓	Tissue	Rahbari et al. 2011 [16] Hu et al., 2018 [12]
miR-296	hsa-miR-296-5p	20q13.32	↓	Tissue	Corbetta et al. 2010 [55]
miR-139	hsa-miR-139-5p	11q13.4	↓	Tissue	Corbetta et al. 2010 [55] Hu et al., 2018 [12]
miR-503	hsa-miR-503-5p	Xq26.3	↑	Tissue	Corbetta et al. 2010 [55]
miR-222	hsa-miR-222-3p	Xp11.3	↑	Tissue	Corbetta et al. 2010 [55] Hu et al., 2018 [12]
miR-517c	hsa-miR-517c-3p	19q13.42	↑ ↓	Tissue Tissue	Vaira et al., 2012 [62] Hu et al., 2018 [12]
miR-27a	hsa-miR-27a-5p	19p13.13	↑	Serum	Wang et al., 2021 [34]
miR-342-3p	hsa-miR-342-3p	14q32.2	↓	Serum	Krupinova et al., 2021 [47]

Decrease in miRNA expression levels (↓); Increase in miRNA expression levels (↑).
